# Programmed Cellular Necrosis Mediated by the Pore-Forming α-Toxin from *Clostridium septicum*

**DOI:** 10.1371/journal.ppat.1000516

**Published:** 2009-07-17

**Authors:** Catherine L. Kennedy, Danielle J. Smith, Dena Lyras, Anjana Chakravorty, Julian I. Rood

**Affiliations:** 1 Australian Bacterial Pathogenesis Research Program, Department of Microbiology, Monash University, Clayton, Victoria, Australia; 2 Australian Research Council Centre for Excellence in Structural and Functional Microbial Genomics, Department of Microbiology, Monash University, Clayton, Victoria, Australia; 3 Department of Biochemistry and Molecular Biology, Monash University, Clayton, Victoria, Australia; Schepens Eye Research Institute, United States of America

## Abstract

Programmed necrosis is a mechanism of cell death that has been described for neuronal excitotoxicity and ischemia/reperfusion injury, but has not been extensively studied in the context of exposure to bacterial exotoxins. The α-toxin of *Clostridium septicum* is a β-barrel pore-forming toxin and a potent cytotoxin; however, the mechanism by which it induces cell death has not been elucidated in detail. We report that α-toxin formed Ca^2+^-permeable pores in murine myoblast cells, leading to an increase in intracellular Ca^2+^ levels. This Ca^2+^ influx did not induce apoptosis, as has been described for other small pore-forming toxins, but a cascade of events consistent with programmed necrosis. Ca^2+^ influx was associated with calpain activation and release of cathepsins from lysosomes. We also observed deregulation of mitochondrial activity, leading to increased ROS levels, and dramatically reduced levels of ATP. Finally, the immunostimulatory histone binding protein HMGB1 was found to be released from the nuclei of α-toxin-treated cells. Collectively, these data show that α-toxin initiates a multifaceted necrotic cell death response that is consistent with its essential role in *C. septicum*-mediated myonecrosis and sepsis. We postulate that cellular intoxication with pore-forming toxins may be a major mechanism by which programmed necrosis is induced.

## Author Summary

*Clostridium septicum* is a highly virulent pathogen that causes spontaneous gas gangrene or clostridial myonecrosis. The essential virulence factor of *C. septicum* is a β-barrel toxin, α-toxin, that forms small pores in host cell membranes. This toxin is frequently described as a hemolysin, because the formation of these pores causes lysis of red blood cell cells due to membrane disruption. However, this description does not recognize additional effects that may be observed in nucleated host cells, which are more sensitive to α-toxin. We investigated how nucleated cells responded to α-toxin by treating a physiologically relevant muscle cell line with purified toxin and monitoring the response using various assays. We observed α-toxin-mediated programmed cellular necrosis that culminated in the release of the immunostimulatory molecule, HMGB1. This mechanism of cell death induction is consistent with the extensive necrosis that is evident in *C. septicum*-mediated myonecrosis and with the overwhelming sepsis that frequently contributes to the high mortality rate. These results represent an important advance in the understanding of the toxicity of β-barrel pore-forming toxins and how they may contribute to necrotic and systemic disease pathology.

## Introduction

*Clostridium septicum* is a Gram-positive anaerobic bacterium that is the primary etiological agent of atraumatic clostridial myonecrosis, a rapidly fulminating and frequently fatal necrotic disease of the human musculature [1]. The primary virulence factor of *C. septicum* is α-toxin, a pore-forming toxin belonging to the aerolysin family of extracellular toxins [2],[3]. *C. septicum* α-toxin is secreted as inactive protoxin monomers that bind to GPI-anchored proteins on the target cell [4]. The bound monomers are then cleaved and activated by host cell proteases [5], allowing them to oligomerize into a heptameric complex and insert to form a 1.6 nm β-barrel pore [6]. Although pore-forming toxins are commonly considered hemolysins due to their lytic effect on erythrocytes, there is evidence to suggest that pore formation may also elicit a broad range of more subtle effects on target cells by initiating signaling pathways [7],[8].

Aerolysin, a well studied ortholog of α-toxin, has been shown to initiate Ca^2+^-mediated apoptosis in T-lymphocytes [9], and G-protein activation and release of Ca^2+^ from intracellular stores in granulocytes [10]. In epithelial cells Ca^2+^ influx was found to inhibit protein kinase B (also known as Akt), which is a key regulator of cell survival pathways [11]. Recently, a novel cell response to aerolysin was reported, namely the caspase-1 dependant repair of cell membranes, which occurs in response to K^+^ efflux [12]. Other small-pore forming toxins, including α-toxin (αHL) from *Staphylococcus aureus* and *Escherichia coli* hemolysin have been reported to elicit a broad range of cellular responses, depending on the concentration of the toxin and the target cell. *E. coli* hemolysin is cytotoxic against a wide range of cell types, with Ca^2+^ influx and ATP depletion frequently observed, contributing to additional downstream effects [13]. αHL has been shown to induce the release of proinflammatory mediators from monocytes and epithelial cell lines [14],[15] and also to induce apoptosis of T lymphocytes [16],[17]. The nature of αHL induced cell death in T-lymphocytes was brought into question when it was shown that while inhibition of caspases prevented DNA laddering and caspase activation in αHL-treated cells, it could not prevent cell death [18]. These data raised the possibility that αHL may also induce a programmed necrosis or oncosis response, as indicated by a rapid depletion of ATP and release of pro-inflammatory histone binding protein high mobility group box 1 (HMGB1) [18]. This divergence from apoptotic cell death is consistent with a growing body of evidence indicating that aside from the ‘classical’ programmed cell death pathway of apoptosis, there is a second poorly characterized ‘programmed necrosis’ or ‘oncosis’ pathway [19],[20], which is “programmed in the sense that it would constitute a stereotyped, evolutionarily designed sequence of biochemical events” [21].

**Figure 1:**
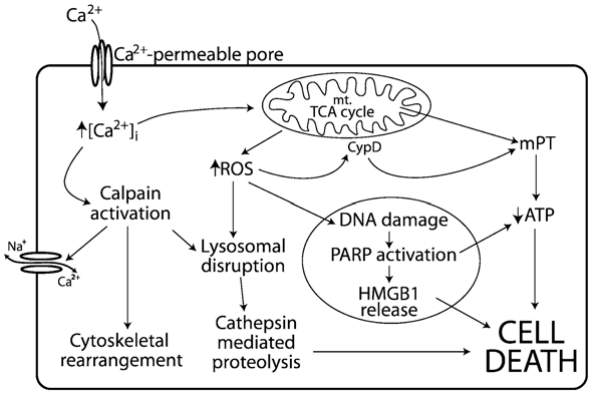
Ca^2+^ mediated programmed necrosis pathways. Increases in [Ca^2+^]_i_ activate Ca^2+^-dependant proteases, which disrupt lysosomes, releasing cathepsins, and cleave cytoskeletal proteins and Na^+^/Ca^2+^ exchange pumps, causing additional Ca^2+^ influx. [Ca^2+^]_i_ increases also disrupt the TCA cycle, leading to increased ROS and mitochondrial permeability transition (mPT), resulting in decreased ATP production. ROS permeabilize lysosomes, acting to further depolarize mitochondria through the activity of cyclophilin-D (CypD) and damage DNA, which activates PARP. PARP depletes ATP and aids the nuclear-cytosolic translocation of HMGB1.

The pathways of programmed necrosis vary considerably, depending on how the initiating insult is recognized by the cell, however, there are some similarities in the morphological changes induced following a necrotic stimulus. Necrosis is characterized by cell swelling, the induction of an inflammatory response, increased intracellular calcium ([Ca^2+^]_i_), massive depletion of ATP and an increase in reactive oxygen species (ROS) [21],[22],[23]. Programmed necrosis can be initiated by a variety of insults; in the context of membrane permeabilization following pore formation it is an increase in [Ca^2+^]_i_ that is the likely progenitor [19],[24] (Figure 1). Increases in [Ca^2+^]_i_, best studied in the context of neuron excitotoxicity [20], can result in the activation of Ca^2+^ dependant proteases, namely calpains, which are then responsible for the degradation of cellular components including Na^+^/Ca^2+^ exchange pumps, the actin cytoskeleton and lysosomes [20],[23]. In this ‘calpain-cathepsin cascade’ lysosomal disruption causes leakage of acidic proteases, some of which retain activity in the neutral cytosol (eg. cathepsins B, D and L) and cause greater proteolytic damage to the cell [25]. Ca^2+^ also contributes to disruption of mitochondrial permeability, leading to a reduction in ATP production and an increase in ROS; the latter can then cause further perturbation of mitochondrial function, lysosomal permeability and DNA damage [20],[26]. DNA damage causes the activation of poly(ADP-ribose) polymerase (PARP), which acts to further deplete ATP [27] and has recently been shown to be involved in the translocation of the immunostimulatory histone binding protein HMGB1 from the nucleus to the cytosol, such that HMGB1 eventually is released following cell lysis [28]. The release of HMGB1 is clinically relevant as it has been shown to be a significant contributor to late sepsis and septic shock [29].

It has been shown that the *C. septicum* α-toxin is considerably more active against nucleated cells than erythrocytes; for example, the murine myoblast C2C12 cell line is 200-fold more sensitive to α-toxin than mouse erythrocytes [30],[31]. In addition, we recently demonstrated that a *C. septicum* strain expressing an α-toxin variant that was able to bind and oligomerize, but not form pores, was avirulent [32]. Together, these data led us to postulate that α-toxin was more active against nucleated cells because they were responding to lower concentrations of toxin by a programmed cell death pathway initiated in response to permeabilization of the plasma membrane. In this paper we demonstrate that *C. septicum* α-toxin induces programmed necrosis in C2C12 myoblasts as a consequence of Ca^2+^ influx following pore-formation, which results in the activation of Ca^2+^-dependant proteases, disturbances to mitochondrial function and release of HMGB1. This form of cell death is consistent with the pathology of *C. septicum*-mediated myonecrosis, which is characterized by extensive muscle necrosis and rapid progression to fulminant sepsis.

## Results

### α-toxin intoxication of mouse myoblasts induces Ca^2+^ influx

**Figure 2:**
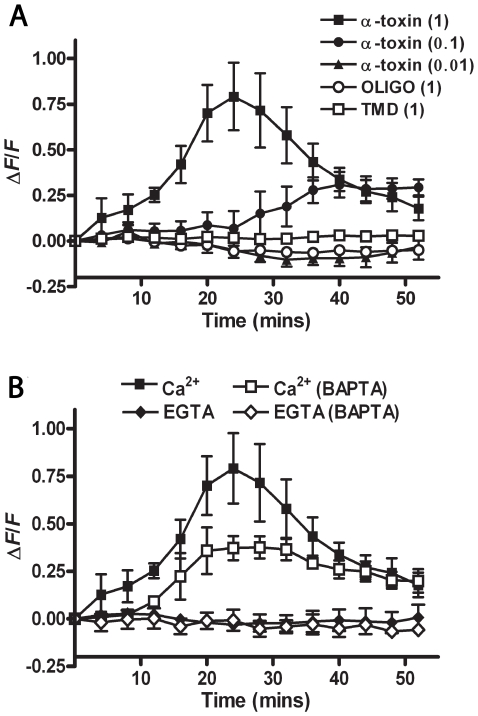
Ca^2+^ influx is dependent on α-toxin pore-formation and extracellular Ca^2+^ availability. (A) C2C12 cells preloaded with the Ca^2+^ reporter fluorophore Fluo 4-AM were treated with purified α-toxin (closed symbols) or mutated derivatives unable to oligomerise (OLIGO) or form a transmembrane pore (TMD) (open symbols) in the presence of 2 mM CaCl_2_. Brackets indicate the concentration of α-toxin in µg/ml. Changes in fluorescence were calculated relative to untreated cells and the starting ratio, as described in Materials and Methods. (B) Changes in [Ca^2+^]_i_ were assessed as for (A) except that C2C12 cells were pretreated with 20 µM BAPTA-AM (open symbols) and/or extracellular CaCl_2_ was replaced with 0.5 mM EGTA. Note that fluorescence was recorded every 2 min, but only data from every 4 min are plotted, for clarity of the figure. Data points represent the mean and standard error from three experiments and all statistical analysis was performed using curves with data at 2 min intervals.

To investigate whether pore-formation by *C. septicum* α-toxin results in intracellular calcium fluctuations, we used the intracellular fluorogenic Ca^2+^ indicator Fluo 4-AM. The murine skeletal myoblast cell line C2C12 was used since *C. septicum*-mediated necrosis occurs predominantly in the skeletal musculature. C2C12 cells pre-loaded with 2 µM Fluo 4-AM were exposed to varying concentrations of purified α-toxin in a buffer containing 2 mM CaCl_2_ and the fluorescence measured at 2 min intervals for 1 h. Intoxication of the cells with α-toxin caused a dose dependant increase in the amount of [Ca^2+^]_i_ compared to untreated cells (Figure 2A). Treatment of cells with mutated toxins, which were either unable to form a transmembrane pore (TMD) or oligomerize (OLIGO) [33],[34] caused no change in [Ca^2+^]_i_ levels (Figure 2A), indicating that pore formation was essential for the changes in [Ca^2+^]_i_.

To confirm these results, we used BAPTA-AM and EGTA, which are intracellular and extracellular Ca^2+^ chelators, respectively. Pretreatment of cells with BAPTA-AM significantly attenuated the increase in [Ca^2+^]_i_ when the cells were treated with 1 µg/ml of α-toxin (*p*<0.001). Replacement of CaCl_2_ in the buffer with EGTA completely abrogated any changes in [Ca^2+^]_i_ (Figure 2B), confirming that changes in the levels of Ca^2+^ in cells treated with α-toxin were due solely to the influx of extracellular Ca^2+^.

### Analysis of cell death response to α-toxin

**Figure 3:**
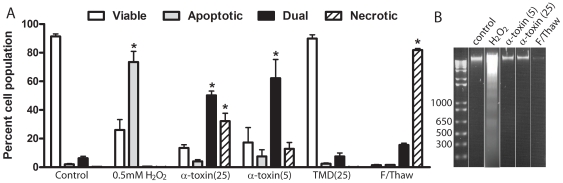
C2C12 cells treated with α-toxin do not exhibit hallmark features of apoptosis. (A) Cells were treated with α-toxin and the TMD deletion derivative at the indicated concentration (ng/ml) for 1 h, apoptosis was induced by treatment with 0.5 mM H_2_O_2_ for 24 h and necrosis by freezing the cells to −70°C and thawing to 37°C. Treated cells were then stained with Annexin V and 7AAD to identify phosphatydylserine exposure indicative of apoptosis, or cell permeability consistent with necrosis, respectively. Dual stained cells were considered late apoptotic/necrotic. The proportion of cells per cell death phenotype was assessed using FACS analysis and the results represent the mean and standard error from three experiments. Note that the lower levels of toxin used here compared to other experiments reflects the low number of cells used per assay. (*) indicates *p*<0.001 compared to the untreated control. (B) Cells were treated as in (A) and internucleosomal DNA was prepared and examined using agarose gel electrophoresis for the presence of 200 bp-fragmentation. Molecular size markers are indicated on the left in bp.

There are reports linking the influx of calcium due to β-barrel pore-forming toxins to the initiation of apoptosis [8],[9] and necrosis [13],[35], therefore we used FACS analysis of Annexin V/7-aminoactinomycin D (7AAD) staining to determine if α-toxin treated cells showed markers of apoptosis or necrosis. Cells that were positive for Annexin V staining alone, indicating the exposure of phosphatidylserine in the absence of cell permeabilization, were considered apoptotic. Cells stained with 7AAD alone were regarded as permeable and therefore necrotic, cells with a dual stained phenotype were considered necrotic or late apoptotic, while unstained cells were deemed viable. Cells were treated with different concentrations of α-toxin; H_2_O_2_ was used as a positive control for apoptosis as C2C12 myoblasts are resistant to more commonly used inducers of apoptosis such as staurosporine and etoposide [36],[37],[38]. Freeze-thaw treatment of cells was used as a positive control for necrotic staining. Analysis of the Annexin V/7AAD staining profiles revealed that α-toxin did not induce apoptosis in C2C12 cells (Figure 3A). Compared to control cells, the proportion of apoptotic cells in the H_2_O_2_ treated sample was significantly increased (*p*<0.001) however, no such increase was observed in α-toxin treated cells. Instead, there was a significant change in the proportion of dual stained cells at both α-toxin concentrations tested (*p*<0.001) and in 7AAD-only stained cells at 25 ng/ml (*p*<0.001). This change was found to be dependent on pore formation since the TMD mutant had no effect on the staining phenotype compared to the control. The lack of induction of apoptosis was confirmed by the absence of internucleosomal DNA fragmentation in α-toxin-treated cells compared to H_2_O_2_-treated cells (Figure 3B). Therefore, it appears that α-toxin induces a necrotic response in C2C12 cells as a result of Ca^2+^ influx.

### The increase in [Ca^2+^]_i_ results in the activation of proteases involved in programmed necrosis

**Figure 4:**
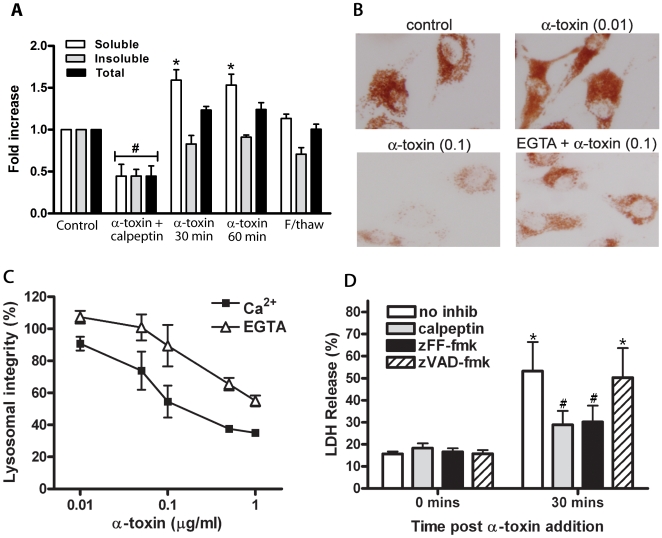
Calpain activation, lysosomal disruption and cathepsin release following α-toxin intoxication of C2C12 cells. (A) Calpain activation was determined in cells that were incubated with 25 ng/ml α-toxin for the indicated times. The detergent soluble and insoluble fractions were assessed for calpain activity using a fluorogenic calpain substrate, and the level of calpain activity determined as a fold change in fluorescence compared to the untreated control. Calpeptin (100 µM) was added prior to toxin incubation (30 min) and substrate cleavage. Bars represent the mean and standard error from three separate experiments. (*) indicates significantly greater calpain activity compared to the respective untreated control (*p*<0.05) and (#) indicates that the calpain activity is significantly less than untreated controls (*p*<0.05). (B) C2C12 cells were treated with neutral red and α-toxin in the presence of either 2 mM CaCl_2_ or 0.5 mM EGTA and assessed for the amount of neutral red retention in the lysosomes by microscopy. Numbers in brackets indicate the concentration of α-toxin in µg/ml. (C) Cells were treated as for (B), then solubilized using 0.5 N HCl/50% ethanol. The amount of neutral red retained was quantified by determining the absorbance at 540 nm, expressed as a percentage of untreated control cells. Data points represent the mean and standard error from three experiments. (D) Cells were treated with calpeptin (100 µM), zFF-fmk (100 µM) or zVAD-fmk (50 µM) for 1 h prior to the addition of 0.1 µg/ml α-toxin. LDH release was used to assess cell death and was expressed as a percentage of the no inhibitor control at 60 min post α-toxin treatment. Bars represent the mean and standard error from three experiments. (*) indicates significantly more LDH release than the respective the 0 time point control (*p*<0.05). (#) indicates that the increase in LDH release compared to the zero time point is not significant.

To assess the downstream effects of the increase in [Ca^2+^]_i_ in intoxicated C2C12 cells we initially focused on the activation of calpains. Calpains are calcium-activated proteases that are important in programmed necrosis because they cause the release of cathepsins from lysosomes and rearrangement of the actin cytoskeleton [39]. C2C12 cells were treated with α-toxin and the detergent soluble and insoluble fractions were assayed for calpain activation using the fluorogenic calpain substrate *N*-Succinyl-Leu-Tyr-7-amido-4-methylcoumarin (*N*-Suc-LY-AMC). We observed a greater than 1.5-fold increase in calpain activity in cells treated with 25 ng/ml α-toxin for 30 and 60 min, compared to untreated cells. Most of the increase was observed in the detergent soluble fraction (*p*<0.05), which suggests that there was an increase in the activity of calpains in the cytosol (Figure 4A). Activity tapered off over the following 4 h, which correlated with a decrease in viability over the same time frame (Figure 4A, data not shown). In agreement with our calcium influx data, neither of the inactive α-toxins was able to induce an increase in calpain activity (data not shown). Specificity was assessed by the addition of the calpain inhibitor calpeptin to the reaction at 100 µM and by subjecting the cells to a freeze-thaw cycle for non-programmed necrosis.

Since calpains are involved in lysosomal disruption [40], we then investigated lysosomal integrity using neutral red. Neutral red is a membrane-permeable supravital dye that becomes impermeable in the acidic environment of intact lysosomes; therefore the integrity of lysosomes can be directly related to the amount of neutral red that is retained by treated cells. A dose dependant reduction in neutral red retention was observed in cells treated with α-toxin in the presence of Ca^2+^, and to a lesser extent in cells treated in the presence of EGTA (Figure 4B). Quantification of this observation confirmed that there was a significant Ca^2+^-dependant reduction in lysosomal integrity, although it was not absolute since a decrease was still observed when the buffer contained EGTA but not Ca^2+^ (Figure 4C).

Since we had now shown that calpains were activated following α-toxin treatment, and that lysosomal disruption had also occurred, potentially leading to leakage of lysosomal cathepsins, we decided to determine the extent to which calpains and lysosomal proteases contributed to cell death. To this end, cells were pretreated with either a calpain inhibitor, calpeptin (100 µM), a cathepsin B and L inhibitor [41], z-Phe-Phe-fluoromethyl ketone (zFF-fmk) (100 µM), or 50 µM z-Val-Ala-Asp-fluoromethyl ketone (zVAD-fmk) to inhibit caspases. Lactate dehydrogenase (LDH) release was assayed to measure the cytotoxicity in response to α-toxin. Both calpeptin and zFF-fmk significantly reduced the amount of LDH released 30 min post α-toxin treatment, compared to the no inhibitor control (*p*<0.05), while zVAD-fmk had no significant effect (Figure 4D). Additionally, pretreatment with calpeptin and zFF-fmk prevented cell death from significantly increasing compared to the zero time point, while both the no inhibitor control and zVAD-fmk treated cells showed significantly more cytotoxicity (*p*<0.01) (Figure 4D). It is important to note that at 60 min after toxin treatment there was no difference in the cytotoxicity observed in untreated cells compared to those treated with calpeptin, zFF-fmk or zVAD-fmk (data not shown). Taken together, these data show that the broad spectrum proteases, calpains and cathepsins, are activated following α-toxin intoxication of C2C12 cells, and that they contribute to the early stages of cell death. However, at later stages of cell death, there appear to be additional factors contributing to cytotoxicity.

### Intoxication of cells with α-toxin causes mitochondrial dysfunction

**Figure 5:**
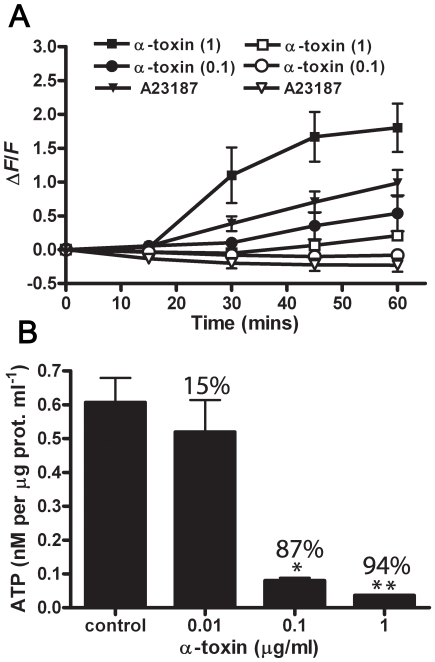
α-toxin treatment mediates mitochondrial dysfunction as indicated by increased ROS levels and depletion of ATP. (A) ROS levels were assessed in cells preloaded with an oxidation sensitive fluorophore following α-toxin intoxication in the presence of extracellular Ca^2+^ (closed symbols) or EGTA (open symbols). The calcium ionophore A23187 (30 µM) was used as a control to confirm the specificity of the Ca^2+^ response. Data points represent the mean and standard error of five experiments; α-toxin concentrations are indicated in brackets. (*) represents *p*<0.01 compared to EGTA control. (B) ATP levels were assessed following α-toxin treatment for 1 h at the indicated concentrations. Values above the bars indicate the percentage ATP depletion compared to the control. Bars represent the mean and standard error of three experiments. (*) represents *p*<0.01 and (**) represents *p*<0.001 compared to the control, respectively.

[Ca^2+^]_i_ increases do not merely have the potential to activate Ca^2+^ dependant proteases. Ca^2+^ overload can also have a direct impact on the function of mitochondria, stimulating the tricarboxylic acid (TCA) cycle, which leads to an increase in the levels of ROS, mitochondrial depolarization and ultimately a severe depletion of ATP [19],[20]. The levels of intracellular ROS in treated and untreated cells were assessed using the probe 2′,7′-dichlorofluorescindiacetate (H_2_DCFDA), which fluoresces upon oxidation. It was shown that ROS levels increased in a Ca^2+^-dependant manner in cells treated with α-toxin (Figure 5A). Ca^2+^ dependence was confirmed by the use of the Ca^2+^ ionophore A23187 and the fact that changes in ROS levels were significantly inhibited when EGTA was used instead of extracellular CaCl_2_. No change in ROS levels was observed in the first 15 min, however, a significant increase was observed after this time point in cells treated with 1 µg/ml of α-toxin, when incubated in the presence of CaCl_2_ compared to EGTA (*p*<0.01).

Apoptosis is considered an energy dependant process, due to the requirement for caspase activation [20], and as such intracellular ATP levels do not significantly decline, at least in the early stages [23]. We quantified the intracellular ATP levels in untreated C2C12 cells compared to cells treated with varying concentrations of α-toxin for 1 h, and found a significant dose-dependent decrease in ATP in α-toxin treated cells (Figure 5B). Moreover, ATP levels decreased to below 85% of normal levels with 0.1 µg/ml of α-toxin, at which point cells are considered necrotic since there is no longer sufficient ATP to maintain energy dependant apoptotic pathways [42]. Taken together, these results suggest that α-toxin-mediated Ca^2+^ influx leads to significant mitochondrial dysfunction, which in turn appears to contribute to a programmed necrosis phenotype.

### HMGB1 release is associated with α-toxin treatment

**Figure 6:**
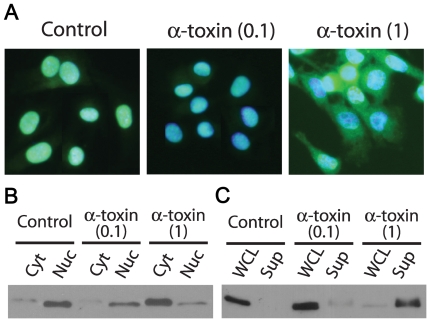
HMGB1 is released into the cytoplasm and supernatant of α-toxin treated cells. (A–B) Intracellular translocation of HMGB1 was visualized using fluorescence microscopy and Western blotting. (A) Cell nuclei were stained with DAPI and HMGB1 localized using anti-HMGB1 antibodies and an Alexa Fluor-488 conjugated secondary antibody. Images are representative of the results from five separate experiments. (B) Western blot analysis of HMGB1 in cytoplasmic (Cyt) and nuclear (Nuc) fractions. (C) Extracellular translocation of HMGB1 was confirmed by Western blot analysis of whole cell lysates (WCL) and culture supernatants (Sup) of α-toxin treated cells compared to untreated control cells. Brackets indicate the concentration of α-toxin in µg/ml.

We then assessed the localization of HMGB1 as additional marker of programmed necrosis. HMGB1 is a chromatin binding protein that in most cell types is selectively retained in the nucleus during apoptosis, but is released into the cytoplasm, and subsequently into the extracellular milieu, during necrosis [43]. Once released from the cell, it acts as a potent mediator of inflammation and cell migration by binding to the Toll-like receptors TLR-2 and TLR-4 and the receptor for advanced glycation end products (RAGE) [19]. We examined the subcellular location of HMGB1 and found that in comparison to untreated cells, where HMGB1 staining co-localized with the nuclei, in cells treated for 1 h with 1 µg/ml of α-toxin, HMGB1 was distributed though the cytoplasm (Figure 6A). No such translocation was identified in cells treated with 0.1 µg/ml α-toxin. These data were confirmed by Western blotting of cytoplasmic and nuclear fractions where most of the HMGB1 was in the nuclear fraction of untreated cells and in the cytoplasm of cells treated with α-toxin (1 µg/ml) (Figure 6B). Since the proinflammatory activity of HMGB1 is dependent on its release from necrotic cells, we also examined the supernatant of α-toxin treated cells. We were only able to detect HMGB1 in the supernatant of cells treated with 1 µg/ml α-toxin, but not in control or 0.1 µg/ml α-toxin-treated cells (Figure 6C). Treatment of C2C12 cells with H_2_O_2_ also led to the release of the protein from cells (data not shown), which is consistent with the fact that H_2_O_2_ causes the passive release of HMGB1 from monocytes and macrophages [44]. The observed translocation of HMGB1 from the nucleus to the cytoplasm and its subsequent extracellular release provides additional evidence that α-toxin causes necrosis in C2C12 cells.

### Differentiated C2C12 cells show the same response to α-toxin

**Figure 7:**
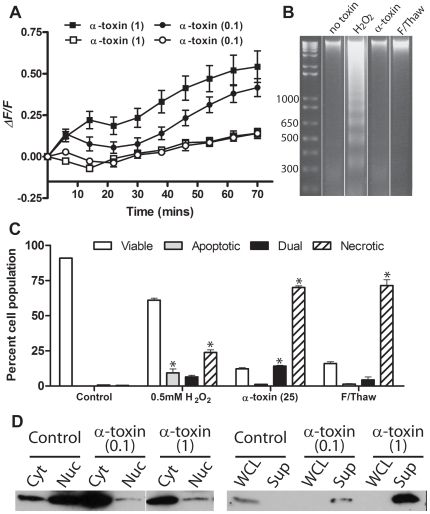
α-toxin treated myotubes display hallmark features of programmed necrosis. (A) C2C12 were differentiated into myotubes and preloaded with the Ca^2+^ reporter fluorophore Fluo 4-AM. Myotubes were treated with purified α-toxin in the presence of extracellular CaCl_2_ (closed symbols) or with 0.5 mM EGTA (open symbols). The levels of [Ca^2+^]_i_ were significantly higher in cells treated with α-toxin in the presence of CaCl_2_ compared to EGTA. Note that fluorescence was recorded every 2 min, but only data from every 8 min are plotted, for clarity of the figure. Data points represent the mean and standard error from three experiments and all statistical analysis was performed using curves with data at 2 min intervals. (B) Internucleosomal DNA was prepared from treated myotubes (25 ng/ml α-toxin, 0.5 mM H_2_O_2_) and examined using agarose gel electrophoresis for the presence of 200 bp-fragmentation. Molecular size markers are indicated on the left in bp. (C) Treated myotubes were stained with Annexin V and 7AAD and the proportion of cells per phenotype was assessed using FACS analysis. The results represent the mean and standard error from three experiments. Note again that the lower levels of toxin used here compared to other experiments reflects the low number of cells used per assay. (*) indicates *p*<0.05 compared to the untreated control. (D) Intracellular translocation of HMGB1. Western blot analysis of HMGB1 in cytoplasmic (Cyt) and nuclear (Nuc) fractions and whole cell lysates (WCL) and culture supernatants (Sup) of α-toxin treated cells compared to untreated control cells. Brackets indicate the concentration of α-toxin in µg/ml.

Since our results indicated that C2C12 myoblast cells responded to α-toxin intoxication by programmed necrosis pathways, we decided to see if these results could be translated into differentiated C2C12 cells, which are a commonly used *in vitro* model of skeletal muscle tissue. Compared to undifferentiated myocytes, C2C12 myoblasts showed a slower influx of calcium at 1 µg/ml, however, the increase was sustained over the course of the experiment (Figure 7A). This change in [Ca^2+^]_i_ was a result of Ca^2+^ influx, since when the buffer was supplemented with 0.5 mM EGTA instead of 2 mM CaCl_2_, no change in intracellular calcium level was observed (Figure 7A). This calcium influx was associated with a necrotic rather than apoptotic phenotype, as determined by FACS analysis of AnV/7AAD stained cells and internucleosomal DNA degradation (Figure 7B and C). We were not able to identify by FACS analysis a significant population of apoptotic myotubes in response to H_2_O_2_ treatment (Figure 7C), but recently published work indicates that oxidative stress is able to induce apoptosis in C2C12 myotubes [45], and we were able to detect DNA laddering (Figure 7B). Finally, differentiated cells also showed evidence of HMGB1 release from the nucleus to the cytoplasm and into the supernatant (Figure 7D).

## Discussion

Although *C. septicum* α-toxin is known to be active against several cell types [5],[31], the effects of the toxin have previously only been investigated in detail in *Toxoplasma gondii* tachyzoites, where it causes membrane perturbations and vacuolization [46]. Our studies have now shown that *C. septicum* α-toxin has the ability to induce a programmed necrosis response in murine myoblast cells and differentiated myotubes; a response that results from an increase in [Ca^2+^]_i_. This result is in contrast to reports of cellular responses to the orthologous toxin, aerolysin [9],[10],[11], however, it must be noted that cellular responses are frequently cell type specific [8].

Intracellular increases in Ca^2+^ concentration are a common feature of many cell responses to bacterial pathogens and are generally associated with activation of the Ca^2+^-dependant protease, calpain, which causes cytoskeletal rearrangement [24], lysosome rupture [40] and cleavage of Na^+^/Ca^2+^ exchange pumps, further increasing [Ca^2+^]_i_ [47]. Ca^2+^ influx is also associated with mitochondrial dysfunction, as a result of Ca^2+^ stimulation of the mitochondrial TCA cycle, leading to the production of ROS and depletion of ATP [20] (Figure 1). Since Annexin V/7AAD staining indicated that α-toxin-mediated cell death appeared to be predominantly late apoptotic and/or necrotic, we focused on identifying biochemical changes that were consistent with a Ca^2+^-induced programmed necrosis phenotype. To this end, we were able to identify activation of both the calpain-cathepsin cascade and mitochondrial dysfunction, leading to ROS production, ATP depletion and ultimately HMGB1 release.

The dual responses of calpain activation and mitochondrial dysfunction, and their intertwined pathways, most likely contributed to the rapid cell death response that was identified. That calpain and cathepsin inhibition could not completely prevent cell death indicated that mitochondrial dysfunction was a significant contributor to cytotoxicity - indeed the drastic increase in ROS and severe ATP depletion could be sufficient to cause cell death [23]. ROS are not only a marker of over stimulation of the TCA cycle, but they can also have a positive feedback effect by causing greater permeabilization of mitochondrial membranes [48], as well as disrupting lysosomes [26]. Using calpeptin as a calpain inhibitor we were unable to prevent lysosomal disruption (data not shown), suggesting that in this model ROS may have been the primary cause of deleterious effects on the lysosomes. ROS are also a major contributor to DNA damage, which in turn leads to the activation of PARP [49]. In healthy cells PARP acts to repair DNA strand breaks; excessive DNA damage and consequent hyperactivation of PARP leads to the exhaustion of ATP stores [27], which would compound the ATP depletion caused by the Ca^2+^-induced mitochondrial dysfunction. In apoptotic cells, significant ATP depletion is prevented by caspase-mediated cleavage of PARP [50]. In support of α-toxin mediating a programmed necrosis cell death response, our data showed that ATP was significantly depleted, to below 85% within an hour of α-toxin treatment, after which point cells are considered irretrievably necrotic [23],[42].

Pore-forming toxins have been reported to initiate apoptosis at low concentrations and necrosis/oncosis at higher concentrations [18],[35]. We were not able to demonstrate α-toxin-mediated apoptosis by either FACS analysis of Annexin V/7AAD differential staining, or by DNA laddering at the toxin concentrations tested, however, α-toxin has been reported to induce DNA laddering in Chinese hamster ovary cells at subnanomolar concentrations [51]; the same concentration required for aerolysin-mediated DNA laddering [9] and much lower than the concentration of α-toxin used in this study. The induction of DNA laddering was only used as a measure of cytotoxicity, and was not characterized further, so it is not known whether the laddering was the result of low level Ca^2+^ influx as shown for aerolysin [9], or depletion of cytosolic K^+^, which α-toxin has also been shown to cause [2], and which is important in the regulation of caspase activation and DNA laddering [52]. An alternative cell death pathway, pyroptosis, was recently coined to describe caspase-1 dependant necrosis [53], which is predominantly associated with bacterial invasion and plasma membrane permeability by type III secretion mechanisms [53], although *Mannheimia haemolytica* has been shown to mediate pyroptosis via its leukotoxin [54]. The *C. septicum* α-toxin-mediated cell death we observed is similar to pyroptosis in that there is no apparent DNA laddering in cells, however, mitochondrial integrity is maintained in pyroptosis [53], whereas it is disturbed in our system. It is concluded that despite similarities with other described cell death pathways α-toxin-mediated cell death in C2C12 myoblasts follows a predominantly necrotic pathway.

Early work published on the activity of pore-forming toxins, before programmed necrosis was described as a biochemical pathway, described cellular responses consistent with the induction of programmed necrosis. αHL mediates Ca^2+^ influx in PMNs [55], and Ca^2+^ influx and ATP depletion in T lymphocytes when applied at high doses [16]. *E. coli* hemolysin, a prototype of the RTX hemolysins, causes Ca^2+^ influx and ATP depletion in a wide variety of target cells [13]. In human monocyte-derived macrophages, enteroaggregative and cell-detaching *E. coli* strains were found to cause hemolysin A-dependent phenotypic changes in cell morphology that are consistent with oncosis [56]. More recent work on pore-forming toxins reveals the involvement of several mediators of programmed necrosis. CaCo-2 cells treated with *Clostridium perfringens* enterotoxin undergo apoptosis or oncosis, depending on the concentration of toxin [35]. Calpain activation was demonstrated and both apoptotic and oncogenic cell death pathways could be prevented by calpain inhibition [35]. At low concentrations, αHL has long been considered to induce apoptosis, as characterized by DNA fragmentation and caspase activation [7], however, while inhibition of caspases prevents the hallmark features of apoptotic cell death, it does not ultimately prevent cytotoxicity [18]. Similar effects were observed in *Streptococcus suis*-infected porcine choroid plexus epithelial cells [57]. Both studies also reported the translocation of HMGB1 from the nucleus, even in the absence of caspase inhibition, indicating the cell death response was predominantly necrotic, despite the observation of apoptotic hallmarks.

The observation that α-toxin induces the release of HMGB1 from muscle cells is a highly significant finding, considering its immunostimulatory properties. HMGB1 has been shown to be a potent mediator of late septic shock that results from endotoxin stimulation of macrophages [29],[58] and there are indications that it may be a valuable therapeutic target in the treatment of sepsis [59]. *C. septicum* infections are frequently fatal, even with aggressive antimicrobial and surgical interventions [60], and patients most commonly die from overwhelming septic shock, despite the localized nature of myonecrosis. Further research is required to better characterize the contribution of HMGB1 to the pathogenesis of *C. septicum* infections and the potential role it plays in disease mediated by other pathogens that produce similar pore-forming toxins.

In summary, we have shown that *C. septicum* α-toxin mediates programmed necrosis of C2C12 murine myoblast cells, necrosis that is characterized by calpain activation, increased levels of ROS, ATP depletion and HMGB1 translocation. The necrotic nature of the cell death response observed in these cells parallels the infectious process of *C. septicum*-mediated myonecrosis, where there is extensive destruction of the skeletal muscle tissue and septic shock [1],[3]. Most data pertaining to Ca^2+^-activated necrosis/oncosis pathways is derived from neuron excitotoxicity [20] and ischemia/reperfusion models [25], and to our knowledge this report is the first to analyze multiple aspects of Ca^2+^-induced necrosis/oncosis in response to a pore-forming toxin. We postulate that pore-forming toxins may form a major class of inducers of the programmed necrosis pathway.

## Materials and Methods

### Cells, reagents and antibodies

C2C12 mouse myoblast cell lines were maintained in DMEM media supplemented with 10 mM L-glutamine and penicillin/streptomycin (Gibco, Invitrogen) and 10% fetal calf serum (MultiSer, Cytosystems, Castle Hill, Australia). Cells were differentiated by culturing to confluency, substituting normal growth media for DMEM supplemented with 2% fetal calf serum and then culturing for a further five days. Protease inhibitors, calpeptin and cathepsin L inhibitor I were purchased from Calbiochem (Merck KgaA, Darmstadt, Germany) and zVAD-fmk from Bachem (AG, Bubendorf, Switzerland). The calpain fluorogenic substrate *N*-Suc-LY-AMC, neutral red and Bioluminescent ATP assay kit were obtained from Sigma (St Louis, MO, USA). Fluorogenic indicators Fluo4-AM and H_2_DCFDA were purchased from Molecular Probes (Invitrogen, Carlsbad, CA, USA) as were goat anti-rabbit IgG Alexa-Fluor 488, BAPTA-AM and DAPI. LDH was assayed using the CytoTox-ONE™ Homogeneous Membrane Integrity Assay purchased from Promega (Madison, WI, USA). Expression vectors containing the histidine-tagged structural genes for *C. septicum* α-toxin and its mutated derivatives were a gift from R. K. Tweten and purified as before using Ni^2+^ affinity and cation exchange columns [61]. The construction of the oligomerisation mutant, S178C:C86A, and 10 amino acid TMD deletion have been previously described [33],[34].

### Determination of intracellular Ca^2+^

For Ca^2+^ influx experiments, C2C12 cells were seeded into a black 96-well tray (Nunclon) at 1×10^4^ cells per well. The medium was removed and replaced with HEPES-buffered saline (HBS; 5 mM KCl, 125 mM NaCl, 6 mM D-glucose, 12 mM MgCl_2_, 25 mM HEPES) containing 2 µM Fluo-4AM with or without 20 µM BAPTA-AM and incubated for 1 h at room temperature with shaking. The cells were then washed three times in HBS and covered with HBS supplemented with either 2 mM CaCl_2_ or 0.5 mM EGTA. Toxins were diluted in supplemented HBS and added to the plate in triplicate immediately prior to measurement. For fluorescence measurements, a Tecan Infinite 200 plate reader was used at an excitation wavelength of 485 nm and emission of 520 nm, and the cells were maintained at 37°C for the duration of the measurements. Data were expressed as a ratio of the untreated control relative to the starting ratio using the following equation:
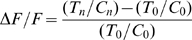
where *F* = the fold change in fluorescence, *T* = average of the readings of the toxin treated replicate samples, *C* = average of the readings of the control replicates, *n* is the time point post toxin addition and *0* is the first reading.

### FACS analysis

C2C12 cells were seeded in six-well tissue culture plates at a density of 2.5×10^4^ cells per well. Cells were treated with 0.5 mM H_2_O_2_ for 24 h or purified *C. septicum* α-toxin at various concentrations for 1 h at 37°C in 5% CO_2_. Adherent C2C12 cells were removed by trypsin treatment, combined with floating cells from the culture medium, washed with 1× PBS and resuspended at 10^6^ cells/ml in 1× Annexin V binding buffer and stained with FITC-conjugated Annexin V and/or 7AAD as per the manufacturer's instructions (BD Biosciences, Heidelburg, Germany). Samples (4000–10,000 events) were acquired and analyzed using a BD Biosciences FACScalibur flow cytometer and CellQuest software.

### Calpain activity

Calpain activity was assessed using a protocol adapted from a previous study [62]. Briefly, 400 µl aliquots of a 1×10^7^ cells/ml suspension of C2C12 cells were treated with 10 ng toxin at 37°C for varying times. Cells were then collected by centrifugation at 400×*g* for 3 min, resuspended in 100 µl of lysis buffer (50 mM Tris-HCl, 0.5% Triton-X, pH 7.3) and incubated on ice for 10 min. The lysate was repeatedly pipetted though a 100 µl protein loading tip to aid the break-up of cells and was then centrifuged at 10,000×*g* at 4°C to separate the detergent soluble and insoluble fractions. The supernatant containing the detergent soluble fraction (crude cytosolic) was removed to a second tube and the detergent insoluble pellet (crude membrane) was resuspended in 100 µl of lysis buffer. The calpain substrate *N*-Suc-LY-AMC was added to a concentration of 10 µM and the fluorescence read at 340/460 nm. Fluorescence intensities corresponding to calpain activity are expressed as a ratio of the treated cells compared to the non-treated control.

### Neutral red retention assay

The neutral red retention assay was adapted from previous studies [63]. Briefly, C2C12 cells were seeded in a clear 96-well tray at 1×10^4^ cells per well. The medium was removed and replaced with serial dilutions of α-toxin in HBS supplemented with 50 µg/ml neutral red (Sigma) and either 2 mM CaCl_2_ or 0.5 mM EGTA, and incubated at 37°C for 1 h. The buffer was then removed and the neutral red taken up by the cells extracted in 50 µl of 0.5 N HCl/50% ethanol for 15 min with shaking at room temperature and the absorbance was read at 540 nm. Lysosomal integrity was calculated as a percentage of the absorbance of the untreated control.

### DNA fragmentation

Internucleosomal DNA was extracted from 1×10^6^ C2C12 cells following α-toxin treatment for 1 h or 0.5 mM H_2_O_2_ for 24 h. Adherent and detached cells were lysed in 0.2% Triton X-100 in TE (10 mM Tris-HCl, pH, 8.0, 1 mM EDTA) and the cell debris and whole nuclei removed by centrifugation at 13 000×*g* for 15 min. The supernatant was then treated with 60 µg/ml RNAse A for 1 h at 30°C, followed by 0.5% SDS and 150 µg/ml proteinase K for 1 h at 50°C. The DNA was then precipitated in 0.1 volumes of 5 M NaCl and 1 volume isopropanol and the entire preparation separated on a 2% agarose gel.

### ROS activation

The levels of intracellular ROS were assayed using the oxidation sensitive fluorogenic reagent H_2_DCFDA. Cells were seeded at 1×10^4^ cells per well in a black 96 well tray. Prior to the assay, the culture medium was removed and replaced with HBS supplemented with 10 µM H_2_DCFDA and the cells were allowed to take up the dye for 30 min at room temperature, with shaking. The cells were then washed three times in HBS to remove unincorporated dye, and the buffer replaced with HBS supplemented with 2 mM CaCl_2_ or 0.5 mM EGTA. Serial dilutions of α-toxin or 30 µM of the Ca^2+^ ionophore A23187 were added immediately prior to reading at 485/520 nm. Calculations of relative fluorescence were performed as for Ca^2+^ measurements.

### Determination of ATP

To determine the levels of intracellular ATP, 1×10^5^ C2C12 cells were treated with varying concentrations of α-toxin for 1 h. Buffer was removed and the cells were resuspended in 200 µl of boiling lysis buffer (100 mM Tris-HCl, pH 7.75, 4 mM EDTA) and boiled for an additional 2 min to inactivate ATPase. Lysates were then centrifuged at 3600×*g* to remove cell debris and the supernatants kept on ice. ATP levels were assayed using an ATP Bioluminescent Assay kit (Sigma), where 100 µl of luciferase reagent mix was added to 100 µl of lysate by automated injection and the luminescence read immediately with 6 sec integration, using a Tecan Infinite 200 plate reader.

### Subcellular fractionation and Western blot analysis

C2C12 cells were treated with varying concentrations of toxin for 1 h. To separate the nuclear and cytosolic fractions, cells were resuspended in lysate buffer (5 mM Tris, pH 7.4, 5 mM KCl, 1.5 mM MgCl_2_, 2 mM EGTA, 1 mM DTT), supplemented with Complete EDTA free protease inhibitor cocktail (Roche Molecular Biochemicals, Mannhein, Germany). Cells were disrupted by repeated vortexing for 15 sec, a sample of the whole cell lysate was collected and fractions were separated by centrifugation at 16,000×*g* at 4°C. The supernatant (cytosolic fraction) was removed and the nuclear fraction was resuspended in buffer containing 10 mM NaCl, 10 mM Tris, pH 7.4, 5 mM EDTA, 1% Triton X-100, supplemented with protease inhibitors as above. For the identification of proteins released into the media, cells were cultured in a minimal volume of media (1% FCS) to effectively concentrate the sample. Samples were standardized to protein concentration using a BCA assay kit (Pierce) and separated on a 12% resolving SDS-PAGE gel before being transferred to a Hybond C+ nitrocellulose membrane. HMGB1 was detected using an anti-HMGB1 antibody (Abcam, Cambridge, UK) at a dilution of 1∶500. The secondary antibody was an anti-rabbit horseradish peroxidase-conjugated antibody (Chemicon International, Temecula, CA, USA) used at a dilution of 1∶1000. Blots were developed by enhanced chemiluminescence (ECL) using the Western Lightning ECL kit (Perkin-Elmer, Boston, MA, USA), according to manufacturer's instructions.

### Immunocytochemistry and microscopy

Cells (5×10^4^ in a 24 well tray) were cultured on glass cover slips and treated as required. To assess neutral red retention, individual cover slips were washed gently in PBS and mounted onto slides immediately prior to observation. To visualize HMGB1 translocation, cover slips were washed three times in PBS and the cells fixed in 3.5% paraformaldehyde in PBS and permeabilized with 0.25% Triton X-100 in PBS. Cells were stained using anti-HMGB1 antibodies (1∶500) and a secondary Alexa Fluor-488 conjugated antibody (1∶200). Cell nuclei were stained using DAPI (0.5 µg/ml) and photographed with an Olympus DP70 camera mounted on an Olympus microscope BX51 using Olysia DP70 software.

### Statistical analysis

Statistical significance was identified using one-way ANOVA, followed by Tukey's post test for multiple comparisons.
